# Endovascular treatment of stroke in the late time window with large vessel occlusion using the ASPECTS as an imaging screening criterion

**DOI:** 10.1038/s41598-024-62936-w

**Published:** 2024-05-29

**Authors:** Jiahui Liu, Na Zhuo, Jianqi Wei, Yu Fan, Changchun Jiang

**Affiliations:** 1 Department of Neurology, Baotou Central Hospital, No. 61 Huanchenglu, Donghe District, Baotou, 014040 Inner Mongolia China; 2 Neurointerventional Medical Center of Inner Mongolia Medical University, Baotou, Inner Mongolia China; 3 Neurological Diseases Clinical Medicine Research Center, Baotou, Inner Mongolia China; 4 Inner Mongolia Medical University, Hohhot, Inner Mongolia China

**Keywords:** Stroke in the late time window; ASPECTS; Large vessel occlusion; Endovascular treatment, Neurology, Medical imaging

## Abstract

To assess the Alberta Stroke Program Early CT Score (ASPECTS) screening tool for effectiveness in endovascular treatment of late time window stroke with large vessel occlusion. A retrospective analysis was performed of individuals administered endovascular treatment in our neurology department between 2016 and 2020 for ischemic stroke induced by acute large vessel occlusion in the anterior circulation and ASPECTS ≥ 6. Detailed baseline and endovascular treatment data were collected. Patients were assigned to 2 groups based on stroke onset time, including the 0–6 h (treated within 6 h of stroke onset) and 6–24 h (earlier/unknown time of onset, up to 24 h from the last time of appearing normal) groups. Both groups were compared for baseline information, revascularization rates, symptomatic intracranial hemorrhage, and 90-day functional independence. Totally 221 individuals were enrolled. The 0–6 h and 6–24 h groups had 129 and 92 patients, respectively, whose median ages were 64 and 63 years, respectively. Both groups were similar in previous medical history, NIHSS score at onset, lesion location and surgical complications. The 6–24 h group had elevated intracranial atherosclerotic stenosis (48.9 vs. 33.3%, *P* = 0.020) and revascularization (96.7 vs. 86.8%, *P* = 0.011) rates versus the 6–24 h group. Upon adjustment for age, sex, National Institutes of Health Stroke Scale, ASPECTS, Intracranial atherosclerosis, intraoperative tirofiban, stent detachment, successful recanalization, and symptomatic intracranial hemorrhage, the 0-6 h group had a higher rate of individuals achieving functional independence (mRS score of 0–2; 52.7 vs. 47.8%, OR = 0.242 [0.070–0.833], *P* = 0.024). However, the rates of individuals with a favorable outcome (mRS scores of 0–3) were similar in both groups (66.7 vs. 69.6%; OR = 0.564 [0.140–2.266], *P* = 0.419) as well as 90-d mortality (OR = 0.889 [0.170–4.660], *P* = 0.889). The ASPECTS is effective for screening individuals for endovascular treatment of stroke in the late time window with large vessel occlusion. The ASPECTS should be considered a simple and practical patient screening strategy for stroke centers without multimodal imaging evaluation.

## Introduction

Endovascular treatment of anterior circulation ischemic stroke has good efficacy and safety profiles within 6 h. Approximately 25% of ischemic strokes occur early in the morning when the patient wakes up and the time of onset is uncertain, i.e., Wake-Up Stroke (WUS)^1^. Because of the uncertainty about the time of onset, such cases are usually excluded from many clinical studies examining acute treatment options for ischemic stroke. The DAWN trial was the first to focus on endovascular treatment (EVT) of WUS and the inclusion criterion was a discrepancy between stroke severity based on the National Institutes of Health Stroke Scale (NIHSS) and baseline infarct volume automatically calculated with the RAPID software^2^. The AURORA meta-analysis demonstrated no heterogeneity in treatment benefit between cases managed in the early and late time windows. Overall, the AURORA study showed a marked therapeutic benefit from endovascular therapy versus standard therapy, resulting in a 2.77-fold increase (95% CI, 1.95–3.94) in improved outcome at 90 days^3^. Most of the studies analyzed in the AURORA analysis used quantitative perfusion software for patient screening. As the RAPID software is not widely available in China, some hospitals rely on CT perfusion imaging, but are disadvantaged by the inability to quantify the penumbra, which makes patient screening challenging. The Alberta Early Stroke Program CT Score (ASPECTS) provides a generally reliable measurement of infarct core assessment in acute ischemic stroke (AIS). Considering clinical symptoms, the presence of a small infarct core along with severe clinical symptoms suggests the presence of an ischemic penumbra that can benefit from EVT^4^. The accuracy of the ASPECTS may improve further with prolonged time to symptom onset and the infarct core becoming more visible on CT. The objective of the present study was to assess endovascular treatment for safety and efficacy in stroke patients with late and unknown time window of up to 24 h after the last normal appearance, identified by the ASPECTS.

## Patient and methods

### Patients

This study analyzed patients administered endovascular therapy for AIS resulting from large vessel occlusion of the anterior circulation between January 2016 and December 2020. EVT were performed following the current guidelines and approved equipment. Inclusion of consecutive patients was based on the following criteria: baseline NIHSS score ≥ 6; internal carotid or proximal middle cerebral artery (M1/M2 segments) occlusion; pre-stroke modified Rankin Scale (mRS) score of 0–2; endovascular treatment started within 24 h of stroke onset. Demographic characteristics, medical history, clinical features, neuroimaging, endovascular treatment procedures were prospectively recorded. mRS scores were assessed at 90 days. Neurologists with extended experience in NIHSS and mRS determined the scores. The Ethics Committee of Baotou Central Hospital approved the research protocol in compliance with the Helsinki Declaration. All patients/legal representatives provided signed informed consent before endovascular therapy.

### Radiological assessment

The brain computed tomography (CT), CT angiography (CTA), CT perfusion (CTP) or magnetic resonance imaging (MRI) were performed at baseline and 24 h. The imaging findings were interpreted by at least two experienced radiologists. ASPECTS assessment considered the final CT image prior to thrombectomy. The ASPECTS scale is a 10-point scoring system that quantifies early ischaemic changes in the middle cerebral artery territory. A score of 10 indicates normal and 1 point is deducted for each abnormal region. In this study, we included patients who presented within 24 h and met the same criteria of an ASPECTS score of 6 or greater, as recommended by guidelines for patients within 6 h of presentation.

### Outcome assessment

Functional independence was the satisfactory outcome at 90 days, reflected by an mRS score of 0–2; a mRS score of 0–3 indicated a favorable outcome. Safety outcomes encompassed symptomatic intracranial hemorrhage (sICH) and any intracranial hemorrhage within 7 days and 90-day mortality following endovascular therapy. SICH was determined according to the Heidelberg criteria as the detection of new intracranial hemorrhage on brain imaging increasing by ≥ 4 or ≥ 2 points in a given NIHSS subgroup, indicating an important change in neurological status.

### Statistical analysis

Patients were assigned to 2 groups based on stroke onset time, including the 0–6 h (treated within 6 h of stroke onset) and 6–24 h (earlier/unknown time of onset, up to 24 h from the last time of appearing normal) groups. Continuous variates presented as median were compared by the Mann–Whitney U test. The χ2 and Fisher exact tests were performed to compare proportions. For between-group comparison of outcomes, multivariable logistic regression analysis was used to calculate the odds ratio (OR) and the corresponding 95% confidence interval (CI). Baseline variates with *P* < 0.1 in univariate analysis were further examined by multivariate analysis. *P* < 0.05 was deemed statistically significant. Data analysis utilized SPSS 22.0 (SPSS, USA).

### Ethics approval

The study was approved by the Medical Ethics Committee of Baotou Central Hospital. (KYLL2022-014).

## Results

**Figure 1:**
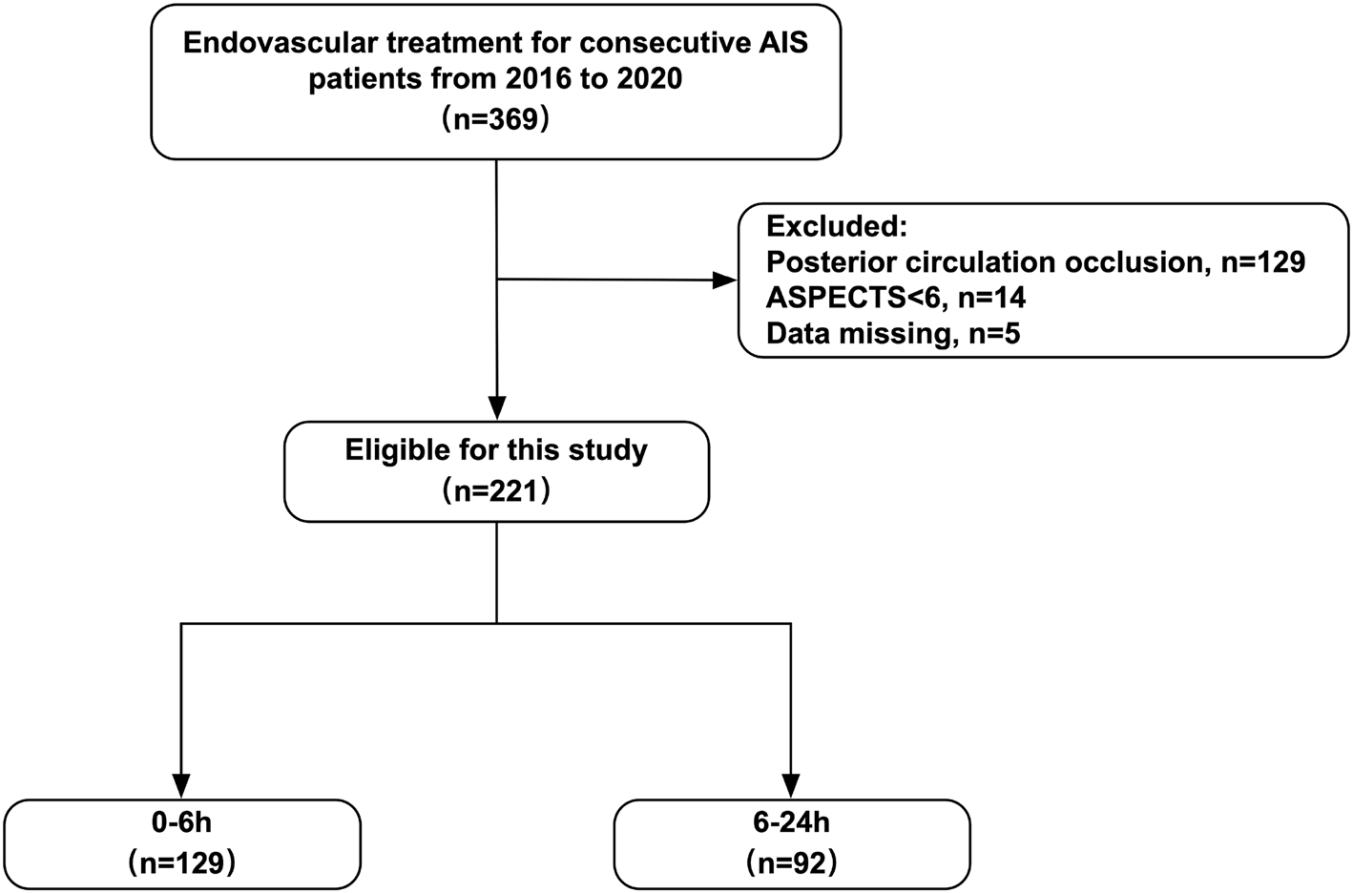
Flow chart of eligible patients.

Totally 369 consecutive patients were enrolled from January 2016 to December 2020 for endovascular treatment of AIS. Of the 221 patients eligible for this study, 129 and 92 were assigned to the 0–6 h and 6–24 h groups, respectively. (Fig. 1) There were no between-group differences in baseline vascular risk factors, symptom onset characteristics, admission NIHSS score, ASITN/SIR score, and ASPECTS (*P* > 0.05), and cases in the 6–24 h group more commonly had concurrent intracranial atherosclerosis (48.9 vs. 33.3%, *P* = 0.020; Table 1).A higher percentage of patients in the 6–24 h group received intraoperative tirofiban (70.5 vs. 47.4%, *P* = 0.020) versus the 0–6 h group, while both groups were similar in the number of stent retriever passes, rescue therapy, procedural complications, immediate and 24-h postoperative NIHSS scores, and puncture to recanalization times. However, an elevated proportion of symptomatic postoperative intracranial hemorrhage was detected in the 0–6 h group versus the 6–24 h group (14 vs. 5.4%, *P* = 0.041; Table 2).

**Table 1:** Baseline information between the 0–6 h onset group and the 6–24 h onset group.

Characteristics	0–6 h (n = 129)	6–24 h (n = 92)	*P* Value
Age (SD)	64 (12)	63 (11)	0.523
Male (%)	56.6	68.5	0.073
Hypertension (%)	54.3	56.5	0.739
Diabetes (%)	20.9	23.9	0.599
Hyperlipidemia (%)	2.3	5.4	0.282
Coronary heart disease (%)	29.5	25	0.465
Atrial fibrillation (%)	31	22.8	0.18
Prior smoking (%)	24.8	23.9	0.879
Current smoking (%)	35.7	39.1	0.598
NIHSS (IQR)	13 (10–15.5)	13 (9–15.75)	0.659
ASITN/SIR (IQR)	1 (0–2)	1 (0–2)	0.458
ASPECTS (IQR)	8 (6–9)	8 (6–8.75)	0.412
With ICAS (%)	33.3	48.9	0.020

**Table 2:** Endovascular treatment information between the 0–6 h onset group and the 6–24 h onset group.

Characteristics	0–6 h (n = 129)	6–24 h (n = 92)	*P* Value
Local anesthesia (%)	92.2	92.4	0.677
Number of Stent retriever passes (IQR)	2 (1–3)	2 (1–3)	0.895
Intraoperative tirofiban (%)	47.4	70.5	0.02
Stent retriever detachment (%)	5.4	12	0.08
Stenting (%)	14.7	22.8	0.123
Balloon expansion (%)	22.5	33.7	0.065
Procedural complications (%)	20.9	20.7	0.96
Successful recanalization (mTICI 2b-3)	86.8	96.7	0.011
Immediate postoperative NIHSS (IQR)	11 (7–14)	10.5 (7–14)	0.762
NIHSS 24 h(IQR)	10 (5–14)	10 (5–14)	0.984
Onset to reperfusion time (IQR)	357 (280–422.25)	530.5 (405.75–677.5)	0.000
Puncture to reperfusion time (IQR)	87.5 (60–125.25)	88.5 (64.75–131)	0.463
Mortality within 90 days (%)	14.7	12	0.553
sICH (%)	14	5.4	0.041

**Figure 2:**
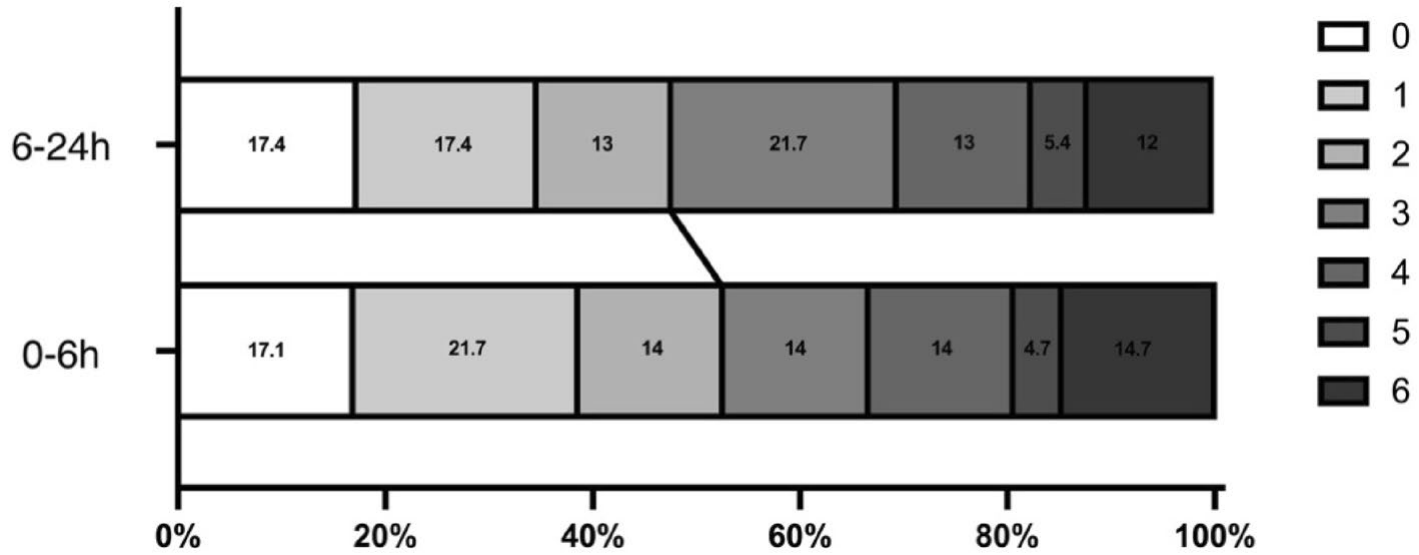
Distribution of 90d mRS in the two groups.

The 90-day functional independence rates were 52.7 and 47.8% in the 0–6 h and 6–24 h groups, respectively. Both groups were comparable in terms of 90-day functional independence, 90-day favorable outcome and 90-day mortality in univariate analysis. Figure 2 shows 90-day mRS scores in both groups. No between-group differences were found after adjustment for age, sex, NIHSS score and ASPECTS. Upon adjustment for age, sex, NIHSS score, ASPECTS, combined intracranial Arterial Stenosis (ICAS), intraoperative use of tirofiban, stent release, successful recanalization, and symptomatic intracranial haemorrhage, the 0-6 h group achieved enhanced functional independence (*P* = 0.024; OR = 0.242, 0.070–0.833), while there were no differences in the favorable outcome and 90-day mortality (Table 3).

**Table 3:** Functional prognosis and safety outcomes.

	0–6 h(n = 129)	6–24 h(n = 92)	Univariate analysis		Multivariate logistic regression			
					Model 1		Model 2	
			OR (95% CI)	*P* Value	OR (95% CI)	*P* Value	OR (95% CI)	*P* Value
Functional independence(mRS 0–2)	52.7	47.8	0.822 (0.481–1.405)	0.47	0.723 (0.397–1.317)	0.29	0.242 (0.070–0.833)	0.024
Favorable outcome(mRS 0–3)	66.7	69.6	1.143 (0.643–2.032)	0.65	1.037 (0.540–1.991)	0.91	0.564 (0.140–2.266)	0.419
Mortality within 90 days (%)	14.7	12.0	0.768 (0.335–1.743)	0.55	0.800 (0.345–1.858)	0.60	0.889 (0.170–4.660)	0.889

Model 1 adjusted for age, sex, NIHSS score and ASPECTS; model 2 adjusted for age, sex, NIHSS score, ASPECTS, combined intracranial Arterial Stenosis (ICAS), intraoperative use of tirofiban, stent release, successful recanalization, and symptomatic intracranial haemorrhage.

## Discussion

The current work revealed that although more patients in the 0–6 h onset group screened by the ASPECTS had a good prognosis (mRS scores of 0–2), both groups had similar rates of patients with a functionally independent prognosis (mRS scores of 0–3) and 90-day mortality rates. These findings indicate that ASPECTS may be a useful clinical screening tool for endovascular treatment in the late window.

The ASPECTS is a clinically useful and cost-effective semi-quantitative tool for assessing the infarct core. Its practicality compensates for the limitations of CTP and PWI, which rely on highly trained technicians or complex image processing for interpretation. Additionally, the ASPECTS is a sensitive and specific measure for predicting the volume of the brain tissue infarct core before revascularization of occluded blood vessels, making it an efficient alternative for time-consuming and intricate processing software^5^. Previous studies have demonstrated ASPECTS and core volume on CTP are moderately correlated^6^. Therefore, the ASPECTS, to some extent, indicates the volume of ischemic brain tissue. The accuracy of ASPECTS assessment increases as infarcts take place over an extended period and as the infarct lesions become more prominent on CT images. In a meta-analysis of the preoperative images of all patients enrolled in five trials, individuals with ASPECTS scores ≥ 6 in the anterior circulation region were shown to significantly benefit from EVT^2,7–10^. Previous studies have shown that although the overall consistency of the ASPECTS score was good, the absolute inter-rater agreement was low. The ASPECTS score in our study was generated by experienced neuroimaging physicians after extensive study. The consistency of the score may be resolved in the future by computer-assisted software.

In clinical practice, only a minority of stroke cases with acute large vessel occlusion arrive at the hospital for treatment within 6 h^11^. It is controversial whether patients with stroke onset of more than 6 h are suitable for EVT, while experience has shown that some of these patients may benefit from EVT. The DAWN and DEFUSE-3 studies utilized quantitative imaging to screen eligible EVT patients and extended the therapeutic window from 16 to 24 h. This widened the time window for EVT, ultimately allowing more patients to benefit from the treatment. The inclusion criteria in both studies were based on CTP infarct volume and ischemic penumbra, quantitatively assessed with the RAPID software. With core infarct < 70 ml and ischemic penumbra ≥ 15 ml, EVT is appropriate when the ischemic penumbra/infarcted core ratio is ≥ 1.8^2,7^. The DAWN and DUFUSE 3 screening criteria are not feasible for appropriate patient screening in most hospitals in China as the RAPID software is not widely accessible in these institutions. The recently published RESCUE-Japan LIMIT study included a subset of individuals with a last known well time of 6–24 h, using no FLAIR signal changes as a criterion for inclusion. The results confirmed individuals with large cerebral infarcts administered endovascular therapy have improved functional outcomes compared with those administered medical treatment alone^12^. The latter study demonstrated DWI-FLAIR mismatch could be employed to screen for patients eligible for endovascular treatment of stroke on wake-up. However, the limited availability of 24-h MRI screening in the majority of Chinese hospitals is a significant obstacle. Nguyen et al. found no marked differences in clinical outcomes in CT-screened patients administered anterior circulation endovascular therapy during the late window period in comparison with those selected for CTP or MRI. These findings support the use of CT for rapid patient screening for endovascular treatment of wake-up stroke or in the late window, enabling patients who cannot undergo CTP to still benefit from endovascular therapy^13^.

Recent studies have shown that endovascular treatment is beneficial for patients with large infarcts and ASPECTS scores of 0–5. Most randomized controlled trials (RCTs) on endovascular treatment for acute ischemic stroke with large core infarcts, such as RESCUE-Japan LIMIT, ANGEL-ASPECTS, and SELECT2, have used advanced imaging techniques (MRI and/or CTP) to screen patients for treatment^12,14,15^. However, numerous hospitals, particularly primary care facilities, lack the capacity to perform advanced imaging on a 24-h basis. The objective of our study was to screen patients with late time windows suitable for mechanical thrombolysis using only non-contrast cranial computed tomography scans. Our results demonstrate the feasibility of this approach. Future studies will aim to determine the feasibility of using the ASPECTS score on non-contrast CT scans to screen patients with large infarcts.

As demonstrated above, a revascularization rate in the 6–24 h group of 96.7% was determined, which was significantly elevated compared with 86.8% in the 0–6 h group (*P* = 0.011). This may be due to the possibility that the 6–24 h group had a higher rate of concurrent intracranial atherosclerosis. The observed higher incidence of ICAS in the 6–24 h group (48.9%, *P* = 0.020) may suggest better collateral circulation, potentially contributing to the elevated reperfusion rates observed. Despite this, multifactorial analysis adjusted for ICAS revealed no significant inter-group differences, emphasizing the need for cautious interpretation of comparative outcomes. Despite higher proportions of patients requiring rescue balloon angioplasty and stent implantation, this group had lower thrombus burden and subsequently displayed a higher revascularization rate. In addition, the incidence of sICH was markedly reduced in the 6–24 h group in comparison with the 0–6 h group (5.4 vs. 14%, *P* = 0.041), which may be explained by the higher rate of individuals in the 0–6 h group with embolism as the cause of stroke.

There were limitations in the current study, including a single-center inpatient population, with some selection bias. Additionally, as patients were screened over an extended period, the development of treatment tools may have potentially impacted patient prognosis. Future prospective registries or randomized controlled trials are warranted to confirm these findings.

## Conclusions

The ASPECTS is effective for screening individuals for endovascular treatment of stroke in the late time window with large vessel occlusion. It can be considered a simple and practical patient screening tool for stroke centers without multimodal imaging evaluation capacity.

## Data Availability

The datasets used and/or analysed during the current study available from the corresponding author on reasonable request.
